# Spatial distribution patterns of soil total phosphorus influenced by climatic factors in China’s forest ecosystems

**DOI:** 10.1038/s41598-021-84166-0

**Published:** 2021-03-08

**Authors:** Jie Zhu, Anchi Wu, Guoyi Zhou

**Affiliations:** 1 South China Botanical Garden, Chinese Academy of Sciences, Guangzhou, 510650 China; 2 College of Resources and Environment, University of Chinese Academy of Sciences, Beijing, 100049 China; 3 Institute of Ecology, School of Applied Meteorology, Jiangsu Key Laboratory of Agricultural Meteorology, Nanjing University of Information Science and Technology, Nanjing, 210044 China

**Keywords:** Forest ecology, Ecology, Ecology

## Abstract

Phosphorus (P) is an important element in terrestrial ecosystems and plays a critical role in soil quality and ecosystem productivity. Soil total P distributions have undergone large spatial changes as a result of centuries of climate change. It is necessary to study the characteristics of the horizontal and vertical distributions of soil total P and its influencing factors. In particular, the influence of climatic factors on the spatial distribution of soil total P in China’s forest ecosystems remain relatively unknown. Here, we conducted an intensive field investigation in different forest ecosystems in China to assess the effect of climatic factors on soil total P concentration and distribution. The results showed that soil total P concentration significantly decreased with increasing soil depth. The spatial distribution of soil total P increased with increasing latitude and elevation gradient but decreased with increasing longitude gradient. Random forest models and linear regression analyses showed that the explanation rate of bioclimatic factors and their relationship with soil total P concentration gradually decreased with increasing soil depths. Variance partitioning analysis demonstrated that the most important factor affecting soil total P distribution was the combined effect of temperature and precipitation factor, and the single effect of temperature factors had a higher explanation rate compare with the single effect of precipitation factors. This work provides a new farmework for the geographic distribution pattern of soil total P and the impact of climate variability on P distribution in forest ecosystems.

## Introduction

Phosphorus (P) has been an indispensable element of Earth’s biological systems since the beginning of life^1,2^ and it is a major limiting nutrient for plant growth and ecosystem development^3,4^. During the past 20 years, numerous studies have revealed that ecosystems are affected by the lack of P resources^5,6^. Recently, a global meta-analysis research showed that there is significant P limitation in aboveground plant production and that the magnitude of the P limitation is driven by ecosystem properties, climate and fertilization regimes^4^. Limited plant growth is known to directly reduce biodiversity and ecosystem productivity^4,7^, change carbon–nitrogen cycles^8^, and affect other ecological processes^9^. It is thus critical to understand the spatial distribution of soil total P and its driven factors to evaluate soil productivity, improve biodiversity, guide nutrient management, and understand biogeochemical cycles.

Soil total P mainly originates from minerals in the lithosphere that are freed by weathering and chemically transformed within the pedosphere to finally enter the food chain in a dissolved form via plant roots^1,2^. The total amount and chemical forms of P change systematically during soil development^10^. In the initial stages, soil P exists mainly as primary minerals such as apatite. In mid-stage soils, the reservoir of primary apatite is diminished, and less-soluble secondary minerals and organic P constitute an increasing fraction of soil P. In developed soils, soil P is partitioned mainly between refractory minerals^11,12^. We know from chrono sequence data, that P accumulation has occurred in the soil over thousands of years during pedogenesis, whereby the distribution of soil total P is driven largely by abiotic and biotic factors^13^. The climate of a particular area changes over decades and centuries, resulting in large-scale biome migrations^14^. Variations in P distribution patterns and P concentrations are both linked to shifts in climate and biome migrations^15^. Thus, further study is required to understand the characteristics of the horizontal and vertical distributions of soil total P and its influencing factors.

During soil development, the spatial distribution of soil total P is mainly affected by the parent material, the biota present, climatic factors and soil biogeochemical processes^16,17^. These factors, which drive the spatial distribution of soil total P differ significantly at local, regional, and global scales^18–20^. For example, a study on black soils in the northeast of China revealed that the vertical distribution of soil total P showed irregular variations in forests^21^. In contrast, another study in the Yingwugou watershed of the Dan River showed that soil total P decreased with soil depth^22^. In addition, climatic factors (e.g., high precipitation and high temperature) also directly or indirectly affect soil total P concentration and distribution by affecting soil properties, plants, and soil microbial activities^15,23,24^. Recently, Hou et al. research found that soil total P decreased significantly with increasing annual mean precipitation and temperature^24^. Liu et al. explored the pattern of plant nitrogen and P stoichiometry and found that soil total P concentrations decreased along with decreasing mean annual temperature^25^. In general, climatic factors drive the spatial distribution of soil total P in natural forest terrestrial ecosystems^20,24^.

**Figure 1:**
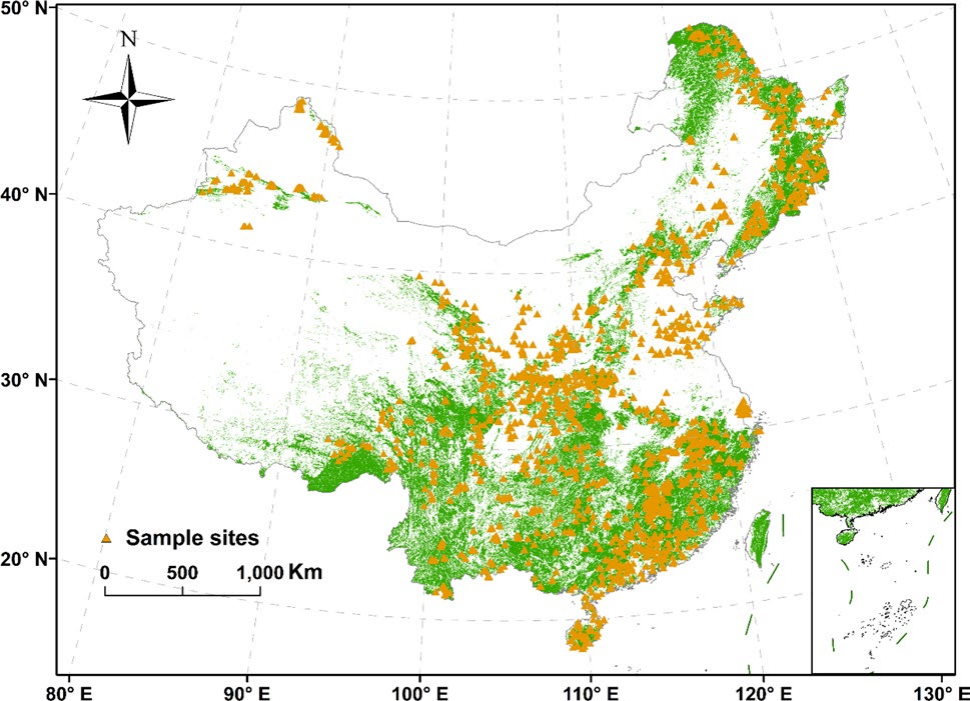
Distribution of sample sites in China. The dark yellow triangles indicate forest plots. (Map created using ArcGIS 10.6 software by first author, URL: http://www.esri.com).

In this study, we performed an intensive field investigation in forest ecosystems of China. We investigated 4293 forest plots and obtained 19 bioclimatic variables to analyze the distribution of soil total P in different soil layers and their relationships with geographic patterns and climate factors. The objectives of this study were: (1) to analyze the spatial distribution pattern of soil total P in China’s froest ecosystem; (2) to quantify the relationship between soil total P and bioclimatic variables; and (3) to explain the main factors affecting the distribution of soil total P. Our final aim was to improve our understanding of the role of climate in shifting soil total P distribution as this knowledge is critical in improving our ability to accurately predict soil total P storage in terrestrial ecosystems (Fig. 1).

## Results

### Vertical distribution pattern of soil total phosphorus concentrations

**Figure 2:**
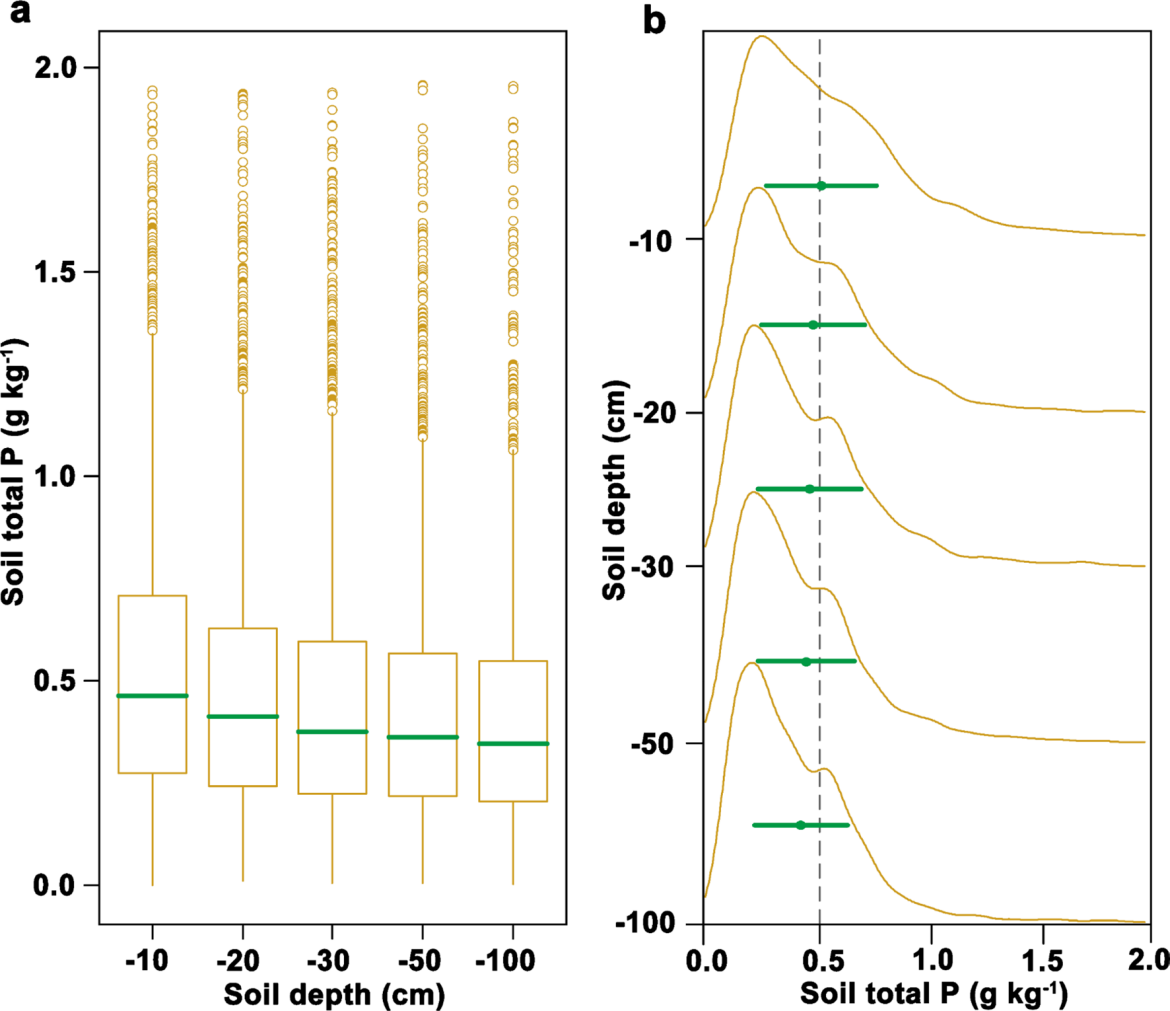
Comparisons of soil total P in terms of its vertical distribution characteristics. (**a**) Soil total P with increasing soil depth. The lines across the boxes indicate the median values, and the lower and upper boxes shows the interquartile range (25th and 75th percentiles, respectively). (**b**) Density distribution of soil total P concentration in the different soil layers. The values of points and error bars indicate means ± SD for the different soil layers. − 10, − 20, − 30, − 50, and − 100 represents the 0–10 cm, 10–20 cm, 20–30 cm, 30–50 cm, and 50–100 cm soil depths, respectively.

Soil total P concentrations significantly decreased with increasing soil depth in China’s forest ecosystems (Fig. 2). The mean concentrations of soil total P in different soil layers were as follows: 0.52 g kg^−1^, 0–10 cm; 0.47 g kg^−1^, 10–20 cm; 0.45 g kg^−1^, 20–30 cm; 0.43 g kg^−1^, 30–50 cm; and 0.40 g kg^−1^, 50–100 cm (Fig. 2a). The standard deviations of soil total P decreased significantly with increasing soil depth, the frequency distributions of lower as well as higher soil total P concentrations decreased gradually with increasing soil depth (Fig. 2b).

### Spatial distribution pattern of soil total phosphorus concentration

**Figure 3:**
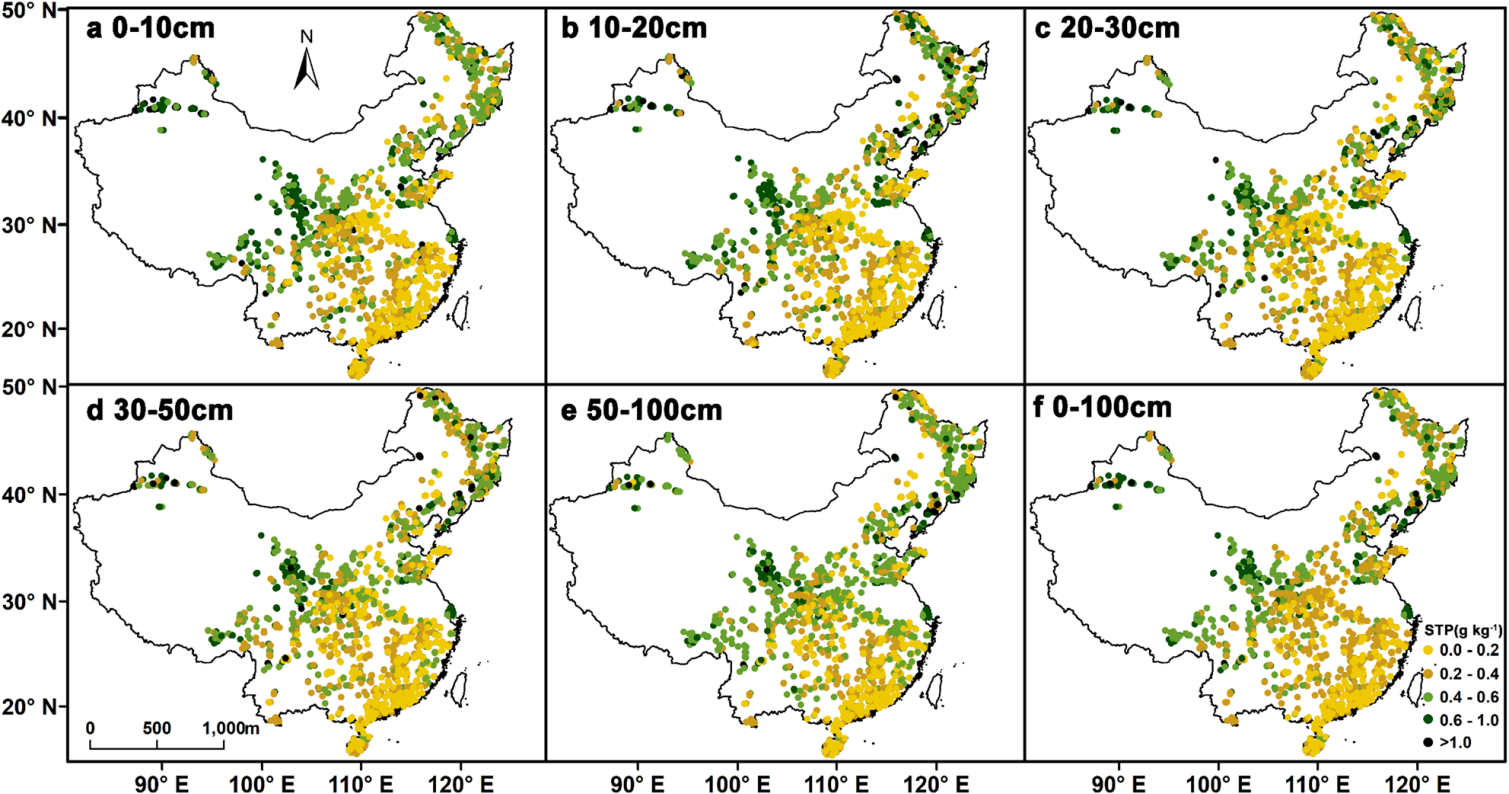
Spatial distribution of soil total P concentrations at different soil depths in China’s forest ecosystems (Map created using ArcGIS 10.6 software by first author, URL: http://www.esri.com).

The spatial distributions of soil total P concentrations across the study region were similar among the different soil layers (Fig. 3). We found that soil total P concentrations generally decreased from north to south (Fig. 3a–e). The highest values were observed in the temperate forest and the edge of Tibetan Plateau. Soil total P concentrations were lowest found in in the tropical and subtropical forests. Moreover, the distribution of soil total P in the 0–100 cm soil depth increment was similar to that observed for the other depth increments (Fig. 3f).

**Figure 4:**
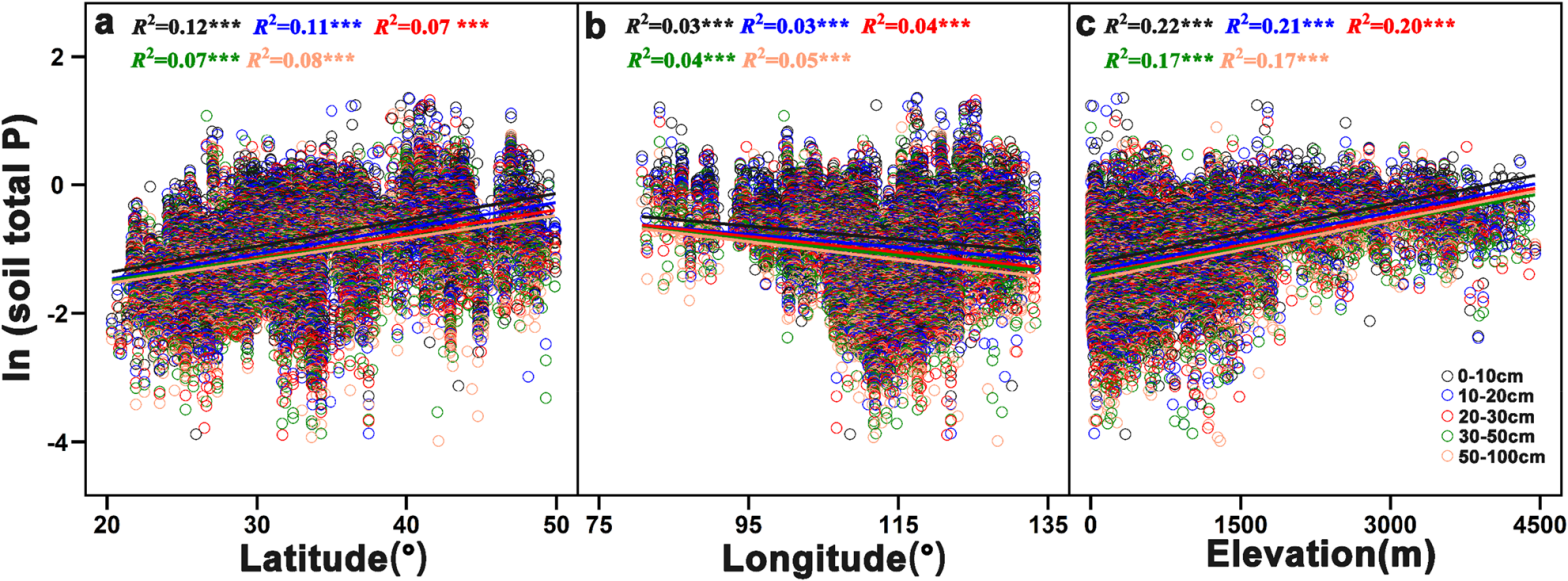
Change in soil total P in China’s forest ecosystems along the latitudinal (**a**), longitudinal (**b**), and elevation (**c**) gradient. *, *p* < 0.05; **p < 0.01; ***p < 0.001.

Our analysis showed that the soil total P in different soil layers significantly increased along both latitudinal and elevational gradient (*p* < 0.001) (Fig. 4a and 4c), whereas it significantly decreased along a longitudinal gradient (*p* < 0.001) (Fig. 4b). Importantly, we further found that the relationship between soil total P and elevation (*R*^2^ value ranging from 0.12 to 0.22) was generally stronger than the relationship between soil total P and latitude (*R*^2^ values ranging from 0.08 to 0.12) or longitude (*R*^2^ values ranging from 0.03 to 0.05) (Fig. 4). Additionally, the *R*^2^ coefficient of linear regression gradually decreased with increasing soil depth in relation to the latitudinal, longitudinal, and elevational gradient (Fig. 4), indicating that the geographic spatial distribution of soil total P varied among the different soil depth increments.

### Effect of climatic factors on soil total phosphorus concentration

**Figure 5:**
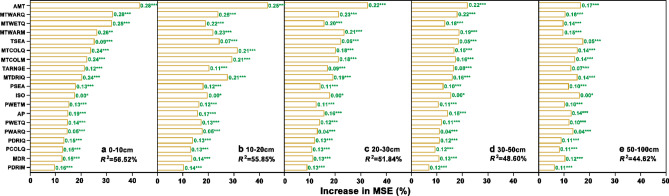
Relative contribution and Pearson’s correlations of climate factors as drivers of soil total P in different soil layers (**a**–**e**). Results from random forest analyses aiming to identify the main temperature and precipitation predictors of soil total P concentration. The increase in the mean square error (MSE, %) is displayed along the x-axes. Pearson’s correlations (green font) indicate the relationships between 19 bioclimatic variables and soil total P in the different soil layers [0–10 cm (**a**), 10–20 cm (**b**), 20–30 cm (**c**) 30–50 cm (**d**), and 50–100 cm (**e**)]. *, *p* < 0.05; **p < 0.01; **p < 0.001.

Regarding soil total P distribution, random forest models revealed that the amount of variation explained by the 19 bioclimatic factors were gradually decreased with soil depth; these factors explained 56.26%, 55.55%, 51.34%, 48.22%, and 44.98% of variation in soil total P in 0–10 cm, 10–20 cm, 20–30 cm, 30–50 cm, and 50–100 cm, respectively (Fig. 5). Furthermore, the relative importance of each variable was also significantly different among the different soil layers. For example, temperature was the most important factor for topsoil, whereas the top five most important factors for the 0–10 cm soil layer were AMT, MTWARD, MTWETQ, MTWARM, and TSEA, respectively (Fig. 5a). The effect sizes of extreme or limiting environmental factors (e.g., PWETQ) generally had a higher impact on soil total P than seasonality (e.g., TSEA) (Fig. 5).

Linear regressions revealed that soil total P had a significant correlation with all bioclimatic factors (*p* < 0.05) (Fig. 5). Note that the strength of the correlations between temperature factors and soil total P significantly decreased with increasing soil depth, but the strength of the correlations between precipitation factors and soil total P changed very little with soil depth (Fig. 5). For example, the Pearson’s correlation coefficient between AMT and soil total P decreased from 0.29 in 0–10 cm to 0.17 in 50–100 cm and the Pearson’s correlation coefficient between AP and soil total P decreased from 0.19 in 0–10 cm to 0.14 in 50–100 cm, indicating that the relative influence of precipitation factors on soil total P gradually increased with soil depth (Fig. 5).

**Figure 6:**
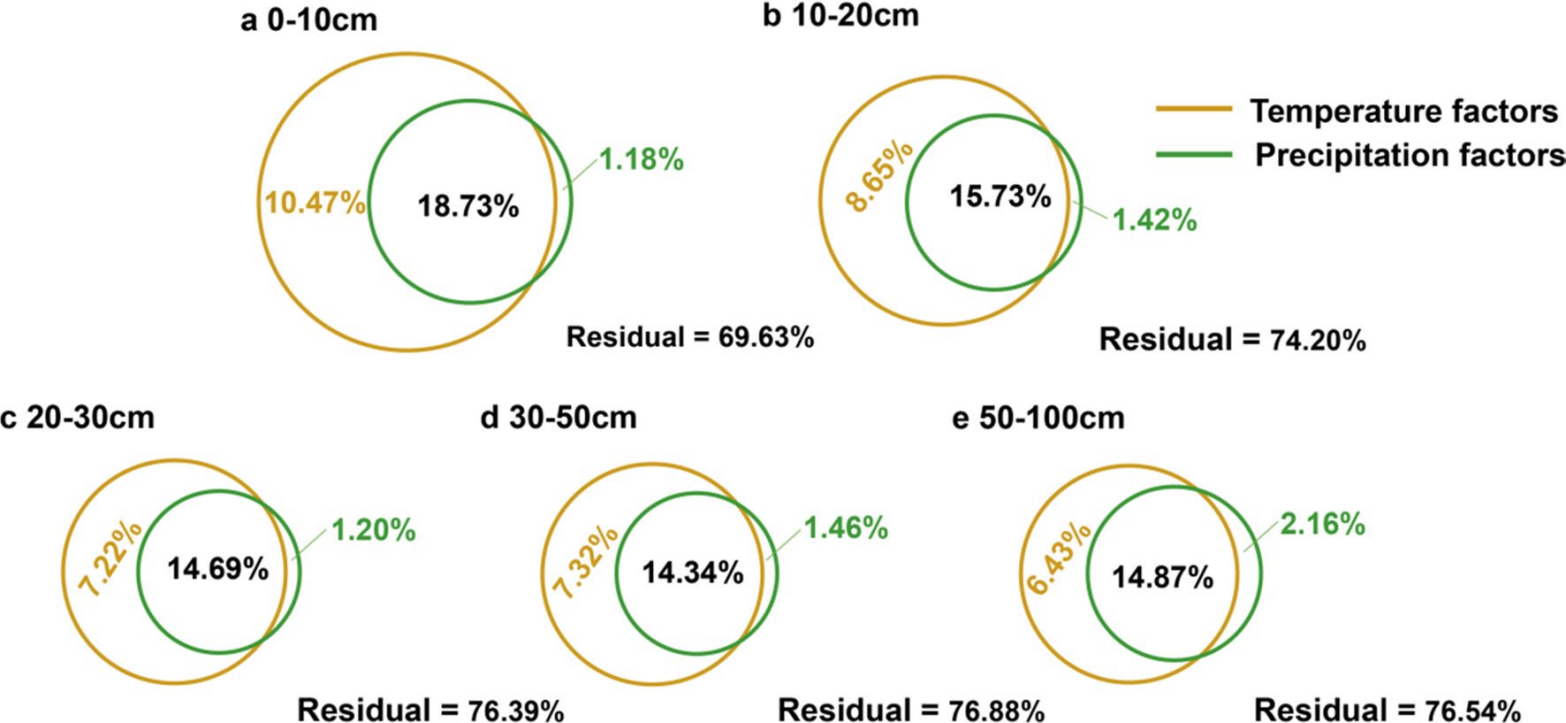
Results of variance partitioning analysis to identity the percentage of variance in soil total P, at different soil depths (**a**–**d**) explained by of temperature and precipitation in China’s forest ecosystems. The sizes of the circles indicate the relative contributions of the factors.

The result of variance partitioning analyses performed to identity the percentage of variance of in the soil total P concentrations that was explained by temperature and precipitation are shown in Fig. 6. Our results indicated that temperature and precipitation factors jointly explained 18.73%, 15.73%, 14.69%, 14.34%, and 14.87% of variation in soil total P at different soil depths (Fig. 6). The single effect of temperature factors explained 10.47%, 8.65%, 7.22%, 7.32%, and 6.43% of the variations in soil total P and the single effect of precipitation factors explained1.18%, 1.42%, 1.20%, 1.46%, and 2.16% of the variations in soil total P at different soil depths. It is noteworthy that the single effect of temperature factors and the combined effects of temperature and precipitation factors gradually decreased with increasing soil depth, whereas the single effect of precipitation factors gradually increased with increasing soil depth (Fig. 6).

## Discussion

Previous studies found that examined the vertical distribution of soil total P concentrations significantly decreased with soil depth across regional scales^21,26,27^. Consistent with this result, we found that soil total P concentrations declined with soil depth across China’s forest ecosystems (Fig. 2), which can be explained by the accumulation of soil total P in the topsoil^28,29^. The large-scale precipitation and temperature on the uppermost soil layer can enhance bedrock and soil chemical weathering rates^23^ and stimulate soil total P accumulation in the topsoil profile^30^. Our results also demonstrated, via random forest models and linear regression analyses, that the effect size and importance of climate factors for topsoil layers were higher than those for subsoil layers. These results further indicate that the climate effect on soil total P distribution is a top-down process and that the accumulation of soil total P is greater in topsoil than in subsoil^31^.

Previous studies have suggested that the vertical distribution patterns of soil total P may also be explained by the effect of biotic and abiotic factors and the multiple pathways resulting in soil layer differences^30^. Soil properties^30^, plant litter inputs^32^, root system secretion^33^, enzyme activities^34^, and microbial decomposition^35^ are known to decrease with increasing soil depth; these factors are ultimately dependent on large-scale climate patterns, because climate affects the distribution of soil total P by controlling the rates of both geochemical weathering and biological activities^30,36^.

Our results showed that soil total P concentration significantly increased with latitude and significantly decreased with longitude. Previous studies conducted at local to global scales have shown inconsistent relationships between soil total P concentrations and latitude or longitude^20,26,37^. This may be due to the global variability in soil age and development, climatic conditions, and topographic heterogeneity^30,37,38^. Soil total P concentration among the different soil depths showed a similar decreasing pattern from north to south in China’s forest ecosystems, which once again indicates that climate is one of the main factors affecting the soil total P distribution at large spatial scales^20,24,39^. The distribution of soil total P showed significant latitudinal and longitudinal trends in our study. This can be attributed to the influence of the Asian monsoon circulations and the Tibetan Plateau topography, which results in China’s temperature and precipitation gradually decreasing from low-latitude tropical regions to mid-latitude cool temperate regions^40^.

Additionally, the geographic distribution of soil total P was affected by altitudinal gradients. Our results showed that the distribution of soil total P significantly increased with elevational gradients, which is in line with most, but not all, previous studies^41,42^. The reason for this increase in soil total P was probably the decrease in soil weathering and/or the increase in soil erosion with altitude, as suggested by the decrease in soil temperature^41^. The change in soil total P concentration with altitude is generally thought to be driven by concurrent changes in temperature^42,43^. Moreover, other factors such as precipitation, the soil parent material, and vegetation types can vary with altitude affecting the distribution of soil total P^42,43^.

The temperature and precipitation variables were negatively correlated with soil total P at the national scale, and the correlation gradually weakened with increasing soil depth. soil total P concentrations increased with decreasing temperature and precipitation variables. The results were in accordance with those previously reported^24^, and may be attributed to a latitudinal gradient of advanced pedogenesis, as soil total P concentration decreases in older and highly weathered soils^44^. In addition, higher plant primary productivity at lower latitudes may be another important reason that the soil total P concentration is lower in hot and wet tropical regions than in cold temperate regions^15^, as plant productivity increases with increasing temperature and precipitation^45,46^. In our study, the influence of temperature and precipitation variables substantially decreased with soil depth. The decline in the correlation between temperature variables and that soil total P concentration was larger than that for the precipitation variables and soil total P along the soil profile from topsoil to subsoil. A possible explanation for this difference is that the decline in soil temperature was larger than the decline in soil water content with increasing soil depth^41,47^.

The effects of temperature and precipitation variables on soil total P distribution have often been mixed (i.e., not well segregated) because of the typical spatial autocorrelation between the two components^39,48^. Our study also showed that the combined effect of temperature and precipitation variables is one of the most important factors affecting soil total P, in agreement with previous studies^24^. The linkage of nutrients to temperature and precipitation factors impacts the variability of soil total P in different soil layers, which can be explained by the fact that temperature can partially counteract the role of additional precipitation by promoting evapotranspiration to affect soil total P distribution^3^. Thus, the result of random forest analysis of temperature factors, precipitation factors suggested that the relationship of soil total P with temperature factors is stronger than that with precipitation factors^15^.

## Conclusions

We evaluated nationwide field data to reveal the distribution of soil total P across forests in China and identified the factors controlling this distribution. Our results showed that the soil total P concentration gradually decreased from north to south. In all soil layers, the concentration was higher in the topsoil than in the subsoil. In terms of its vertical distribution soil total P gradually decreased with soil depth. Soil total P was significantly different among the different regions in our study, and the highest soil total P concentrations were predominantly distributed in the temperate forest and the edge of Tibetan Plateau. In addition, soil total P significantly increased along increasing latitudinal and altitudinal gradients across all soil depths considered but significantly decreased along an increasing longitudinal gradient. Climate greatly affected the distribution of soil total P. The amount of variation explained by19 bioclimatic factors gradually decreased with increasing soil depth, and amount of variation explained by temperature and precipitation converged along the soil profile to become approximately equal in the deepest soil layer we examined. The effect sizes of extreme or limiting environmental factors were higher than those of seasonality factors, and the combined effect of the temperature and precipitation variables was one of the most important factors affecting soil total P distribution. Overall, improving the understanding of the horizontal and vertical distribution of soil total P the factors influencing these distributions is of great significance in the study of nutrient cycles to efforts to improve the sustainable utilization of soil nutrients.

## Materials and methods

### Sample design and soil samples

We completed forest plot selection (at a nationwide scale), soil sampling investigation, and the determination of soil total P from 2011 to 2015, in accordance with a standard protocol^49,50^. Detailed information on the forest plots and soil samples were provided in Tang et al. (2018)^51^. In short, based on the integrity of the soil samples and the distribution of China’s forests, 4293 forest plots were selected (Fig. 1). At each forest plot, we used a soil auger to sample the soil profile to a depth of 1 m, and divided it into five layers: 0–10 cm, 10–20 cm, 20–30 cm, 30–50 cm, and 50–100 cm. Soil samples were then collected from the 0–10 cm soil depth in 4214 plots, 10–20 cm soil depth in 4202 plots, 20–30 cm soil depth in 4062 plots, 30–50 cm soil depth in 3766 plots, and 50–100 cm soil depth in 3121 plots. Soil samples were air-dried, roots and other plant materials were removed, and soil was passed through a 100–mesh sieve prior to elemental analyses. Soil total P concentrations were measured at the wavelength of 700 nm in an ultraviolet spectrophotometer (Lambda 25, Perkin Elmer, Singapore) after H_2_SO_4_-HCIO_4_ digestion.

### Climate data

Climate data for all sites were obtained from Climatologies at high resolution for the Earth’s land surface areas (CHELSA)^52,53^ (http://chelsa-climate.org/). We selected 19 bioclimatic variables, including 11 temperature variables—annual mean temperature (AMT), mean diurnal range (MDR), isothermality (ISO), temperature seasonality (TSEA), maximum temperature of warmest month (MTWARM), minimum temperature of coldest month (MTCOLM), temperature annual range (TRANGE), mean temperature of wettest quarter (MTWETQ), mean temperature of driest quarter (MTDRIQ), mean temperature of warmest quarter (MTWARQ), mean temperature of coldest quarter (MTCOLQ)—and eight precipitation variables—annual precipitation (AP), precipitation of wettest month (PWETM), precipitation of driest month (PDRIM), precipitation seasonality (PSEA), precipitation of wettest quarter (PWETQ), precipitation of driest quarter (PDRIQ), precipitation of warmest quarter (PWARQ), and precipitation of coldest quarter (PCOLQ).

### Statistical analyses

*Soil total P distribution pattern* We used data for soil samples (4214 from 0 to 10 cm, 4202 from 10 to 20 cm, 4062 from 20 to 30 cm, 3766 from 30 to 50 cm, and 3121 from 50 to 100 cm soil depths) to compare the spatial distribution of soil total P among the different soil layers. ArcGIS 10.6 was used to determine and map the pattern in soil total P distribution for the different soil layers.

*General linear modeling* We used simple linear regressions to test the relationships between soil total P concentration and latitude, longitude, and altitude gradients. We also performed Pearson correlations between soil total P and the 19 bioclimatic variables. The statistical significance level was set to *p* < 0.05.

*Random forest modeling* We conducted a classification random forest analysis to identify the main temperature and precipitation actors that predicted the soil total P concentration. Random forest analysis allowed us to identify the most important drivers of soil total P among the 11 temperature and eight precipitation variables. Random forest analysis alleviates multicollinearity problems in multivariate analyses by building bagged tree ensembles and including a random subset of features for each tree (9999 trees). These analyses were conducted using the randomForest package^54^ in R version 3.5.2 (R Core Team (2018) (http://www.R-project.org/)).

*Variation partitioning modeling* The main goal of this analysis was to quantify the relative importance of temperature and precipitation bioclimatic variables at different periods as predictors of soil total P concentrations. To compare the relative effects of the 11 temperature and eight precipitation variables in shaping the spatial distribution of soil total P, we separated the single effect and combined effect of each variable using a Venn diagram^55^ that numbered the relative contribution of each variable to soil total P concentrations in different soil layers. In all cases, variation partitioning analyses were conducted with the “Varpart” function in the R vegan package^56^.
